# The Mitochondria-Targeting Agent MitoQ Improves Muscle Atrophy, Weakness and Oxidative Metabolism in C26 Tumor-Bearing Mice

**DOI:** 10.3389/fcell.2022.861622

**Published:** 2022-03-22

**Authors:** Fabrizio Pin, Joshua R. Huot, Andrea Bonetto

**Affiliations:** ^1^ Department of Anatomy, Cell Biology and Physiology, Indiana University School of Medicine, Indianapolis, IN, United States; ^2^ Simon Comprehensive Cancer Center, Indiana University School of Medicine, Indianapolis, IN, United States; ^3^ Indiana Center for Musculoskeletal Health, Indiana University School of Medicine, Indianapolis, IN, United States; ^4^ Department of Surgery, Indiana University School of Medicine, Indianapolis, IN, United States; ^5^ Department of Otolaryngology-Head and Neck Surgery, Indiana University School of Medicine, Indianapolis, IN, United States

**Keywords:** muscle, cachexia, cancer, mitochondria, metabolism, MitoQ

## Abstract

Cancer cachexia is a debilitating syndrome characterized by skeletal muscle wasting, weakness and fatigue. Several pathogenetic mechanisms can contribute to these muscle derangements. Mitochondrial alterations, altered metabolism and increased oxidative stress are known to promote muscle weakness and muscle catabolism. To the extent of improving cachexia, several drugs have been tested to stimulate mitochondrial function and normalize the redox balance. The aim of this study was to test the potential beneficial anti-cachectic effects of Mitoquinone Q (MitoQ), one of the most widely-used mitochondria-targeting antioxidant. Here we show that MitoQ administration (25 mg/kg in drinking water, daily) *in vivo* was able to improve body weight loss in Colon-26 (C26) bearers, without affecting tumor size. Consistently, the C26 hosts displayed ameliorated skeletal muscle and strength upon treatment with MitoQ. In line with improved skeletal muscle mass, the treatment with MitoQ was able to partially correct the expression of the E3 ubiquitin ligases *Atrogin-1* and *Murf1.* Contrarily, the anabolic signaling was not improved by the treatment, as showed by unchanged AKT, mTOR and 4EBP1 phosphorylation. Assessment of gene expression showed altered levels of markers of mitochondrial biogenesis and homeostasis in the tumor hosts, although only *Mitofusin-2* levels were significantly affected by the treatment. Interestingly, the levels of *Pdk4* and *CytB*, genes involved in the regulation of mitochondrial function and metabolism, were also partially increased by MitoQ, in line with the modulation of hexokinase (HK), pyruvate dehydrogenase (PDH) and succinate dehydrogenase (SDH) enzymatic activities. The improvement of the oxidative metabolism was associated with reduced myosteatosis (*i.e.*, intramuscular fat infiltration) in the C26 bearers receiving MitoQ, despite unchanged muscle LDL receptor expression, therefore suggesting that MitoQ could boost β-oxidation in the muscle tissue and promote a glycolytic-to-oxidative shift in muscle metabolism and fiber composition. Overall, our data identify MitoQ as an effective treatment to improve skeletal muscle mass and function in tumor hosts and further support studies aimed at testing the anti-cachectic properties of mitochondria-targeting antioxidants also in combination with routinely administered chemotherapy agents.

## Introduction

Cancer cachexia is a complex multifactorial syndrome associated with the onset of different types of cancer (Fearon et al., 2011). Its progression debilitates patients, reduces physical function and tolerance to the chemotherapy treatments, impairs quality of life and shortens survival. Prognosis and survival of cancer patients are affected by the presence of cachexia and up to 30% of all cancer deaths will occur as a consequence of cachexia (Fearon et al., 2012). It is estimated that up to 80% of patients with advanced cancer will develop highly debilitating musculoskeletal dysfunctions (von Haehling and Anker, 2014; Sun et al., 2015), and these complications have been reported to persist for months or even years after cancer remission (Hayes et al., 2005; Meeske et al., 2007). Despite its impact, cachexia remains an understudied area of research, and no approved therapies are yet available in the US. The most visible feature of the cachectic phenotype is the progressive body weight loss, accompanied by wasting of skeletal muscle and adipose tissue (Fearon et al., 2011). In particular, muscle atrophy is one of the most concerning aspects of this syndrome, especially since the loss of muscle mass reduces the tolerance to the chemotherapy agents and leads to discontinuation of treatments (Dewys et al., 1980), contribute to the metabolic alterations (Collins et al., 2002; Sanders and Tisdale, 2004; Busquets et al., 2005), leads to physical weakness and severely reduces the performance of daily activities (Hayes et al., 2005; Luctkar-Flude et al., 2009). The pathogenesis of muscle wasting in a cancer setting is a complex phenomenon that involves, among others, increased protein breakdown due to hyperactivation of the proteasome-dependent proteolytic system (Baracos et al., 1995) and of the lysosomal autophagic system (Penna et al., 2013; Aversa et al., 2016), with or without reduced protein anabolism (Kim et al., 2021). Several indications suggest that the muscle regenerative program is also altered in skeletal muscle during cancer cachexia (Penna et al., 2010; He et al., 2013). In addition, impairments of the mitochondrial structure, biogenesis, turnover and metabolism are often detected and can contribute to the energy inefficiency characterizing this syndrome (VanderVeen et al., 2017).

Along this line, evidence of mitochondrial ultrastructural alterations was described in the skeletal muscle of Lewis Lung Carcinoma (LLC) and Colon-26 (C26) carcinoma hosts (Shum et al., 2012; Pin et al., 2015). Such structural changes can be explained by altered mitochondria homeostasis, which we and others reported in the skeletal muscle of tumor hosts (Ballaro et al., 2019; Huot et al., 2020b). In addition, mitophagy, a process regulating mitochondrial turnover, was described increased in cachectic skeletal muscle (Penna et al., 2019). These structural alterations often associate with reductions of the oxidative capacity, as suggested by changes in the activity of the succinate dehydrogenase (SDH) and the pyruvate dehydrogenase (PDH), two important enzymes regulating the TCA cycle, along with modulation of the pyruvate dehydrogenase kinase (PDK)-4, involved in the control of cellular energy metabolism (Pin et al., 2015; Pin et al., 2019a; Pin et al., 2019b). Altogether, these changes contribute to an oxidative-to-glycolytic shift in muscle fiber composition in cachectic tumor hosts *vs.* healthy controls (Pin et al., 2015; Huot et al., 2020a), and participate in the onset of myosteatosis (*i.e.*, the accumulation of intramuscular fat) in cachectic mice (Huot et al., 2020a). Additionally, mitochondrial dysfunctions were reported to increase the levels of oxidative stress and ROS production, thus further promoting mitochondrial damage and degeneration (Penna et al., 2020), which were shown to precede and contribute to muscle atrophy in cancer (Brown et al., 2017). Altogether, these data highlight the importance of preserving and maintaining mitochondrial homeostasis and function to preserve skeletal muscle in cancer and suggest that targeting mitochondria may represent a promising therapeutic intervention against cachexia.

Several drugs have been tested to improve mitochondrial function and energy metabolism (Penna et al., 2018). Some of these compounds specifically exert their function at mitochondrial level and serve as mitochondria-targeting agents endowed with antioxidant properties. In particular, MitoQ and SkQ1 are antioxidants conjugated with lipophilic cations able to accumulate in the mitochondrial membrane due to the difference in membrane potential, whereas the SS-31 peptide accumulates within the mitochondria by binding to cardiolipin (Broome et al., 2018). Interestingly, a recent study showed that administration of SS-31 improved the cachectic phenotype in animals bearing C26 tumors or exposed to chemotherapeutics (Ballaro et al., 2021), whereas Guigni *et al.* reported that the easily accessible MitoQ was able prevent the loss of myosin in myotube cultures exposed to chemotherapy, thus suggesting that it could represent a promising strategy to counteract cachexia also in a setting of cancer (Guigni et al., 2018).

In the present study we validated MitoQ as a tool to prevent mitochondrial alterations and improve the cachectic phenotype in C26 tumor-bearing mice. Our data suggest that chronic administration of MitoQ partially protects against body weight loss, muscle atrophy and weakness following the development of a tumor. These observations are further corroborated by the improvement of the oxidative metabolism in skeletal muscle.

## Methods

### Cell Culture

Murine C26 colon adenocarcinoma cells were provided by Donna McCarthy (Ohio State University, Columbus, OH, United States) and cultured in high-glucose (4.5 g/L) DMEM supplied with 10% fetal bovine serum, 1% glutamine, 1% sodium pyruvate, and 1% penicillin and streptomycin. Cells were maintained in a 5% CO_2_, 37°C humidified incubator. Murine C2C12 skeletal myoblasts (ATCC, Manassas, VA) were grown in high glucose DMEM supplemented with 10% FBS, 100 U/ml penicillin, 100 mg/ml streptomycin, 100 mg/ml sodium pyruvate, 2 mM L-glutamine, and maintained at 37°C in 5% CO_2_. Myotubes were generated by exposing the myoblasts to DMEM containing 2% horse serum (*i.e.*, differentiation medium, DM), and replacing the medium every other day for 5 days. To determine the effect on myotube size dependent on MitoQ (MitoQ Limited, Auckland, New Zealand), myotubes were exposed to 20% C26 conditioned medium (CM) in combination with 75 µM MitoQ (C26 + MitoQ) or triphenylphosphonium cation (TPP, used as non-specific, inactive compound; C26 + TPP) for up to 48 h. Control myotubes were exposed to the same percentage of unconditioned media (UM).

### Animals

All animal studies were approved by the Institutional Animal Care and Use Committee at Indiana University School of Medicine and complied with the National Institutes of Health Guidelines for Use and care of Laboratory Animals and with the 1964 Declaration of Helsinki and its later amendments. All animals were maintained on a regular dark-light cycle (light from 8 a.m. to 8 p.m.), with free access to food and water during the whole experimental period. For the experiments, CD2F1 male mice (Envigo, Indianapolis, IN) were used and housed in a pathogen-free facility at Indiana University Laboratory Animal Resource Center (up to 4 per cage). When the mice were 11 weeks of age, 1 × 10^6^ C26 cells were inoculated subcutaneously (s.c.) in sterile saline. Mice were randomized into four groups: mice receiving TPP (TPP; *n* = 4), mice treated with MitoQ (MitoQ; *n* = 4), C26 tumor-bearing mice receiving TPP (C26 + TPP; *n* = 6) and C26 tumor-bearing mice treated with MitoQ (C26 + MitoQ; *n* = 7). MitoQ was administered in drinking water at 200 μM concentration for the entire duration of the study, starting 2 weeks before tumor inoculation (*i.e.*, at 9 weeks of age). Keeping in mind that each animal generally drinks ∼5 ml of solution per day (data not shown), this dosing equals approximately a 25 mg/kg dosing, in line with previous reports (Ghosh et al., 2010). The mice were monitored for the entire duration of the experiments. After 13 days from tumor injection the mice were sacrificed under light anesthesia (5% isoflurane in oxygen). Several tissues were collected, weighed, snap frozen in liquid nitrogen and stored at −80°C for further analyses. The tibialis anterior muscle was frozen in liquid nitrogen-cooled isopentane, mounted in OCT and stored for morphological analyses.

### Grip Strength

The evaluation of the whole-body strength in mice was assessed as previously described (Bonetto et al., 2015). The absolute grip strength (peak force, expressed in grams) was recorded by means of a grip strength meter (Columbus Instruments, Columbus, OH). Five measurements were completed, and the top three measurements were included in the analysis. In order to avoid habituation, the animals were tested for grip strength at baseline (day 0) and at time of sacrifice (day 13). The investigators were not blinded during the testing.

### Assessment of Muscle Cross Sectional Area

To assess skeletal muscle atrophy, 10-μm-thick cryosections of tibialis anterior muscles taken at the mid-belly were processed for immunostaining as described previously (Huot et al., 2020b). Briefly, sections were blocked for 1 h at room temperature and incubated overnight at 4°C with a dystrophin primary antibody [1:50, #MANDRA1 (7A10), Developmental Studies Hybridoma Bank, Iowa City, Iowa, United States], followed by a 1 h secondary antibody (AlexaFluor 555, 1:1,000, A21127, Thermo Fisher Scientific) incubation at room temperature. Entire dystrophin-stained sections were analyzed for CSA using a Lionheart LX automated microscope (BioTek Instruments).

### Assessment of Myotube Size

C2C12 cell layers were fixed in ice-cold acetone-methanol and incubated with anti-myosin heavy chain antibodies (MF-20, diluted 1:200; Developmental Studies Hybridoma Bank, Iowa City, IA) and an AlexaFluor 488-labeled secondary antibody (Invitrogen, Grand Island, NY). Analysis of myotube size was performed by measuring the minimum diameter of long, multi-nucleate fibers avoiding regions of clustered nuclei on a calibrated image using the ImageJ 1.43 software. Three biological replicates were used for each experimental condition. The results of each replicate were then averaged to obtain the final myotube size.

### Western Blotting

Total protein extracts were obtained by lysing cell layers or homogenizing 100 mg gastrocnemius muscle tissue in radioimmunoprecipitation assay (RIPA) buffer [150 mM NaCl, 1.0% NP-40, 0.5% sodium deoxycholate, 0.1% sodium dodecyl sulfate (SDS), and 50 mM Tris, pH 8.0] completed with protease (Roche, Indianapolis, IN, United States) and phosphatase (Thermo Scientific, Rockford, IL, United States) inhibitor cocktails. Cell debris were removed by centrifugation (15 min, 14,000 g), and the supernatant was collected and stored at −80°C. Protein concentration was determined using the bicinchoninic acid (BCA) protein assay method (Thermo Scientific). Protein extracts (30 μg) were then electrophoresed in 4–15% gradient SDS Criterion TGX precast gels (Bio-Rad, Hercules, CA, United States). Gels were transferred to nitrocellulose membranes (Bio-Rad). Membranes were blocked with SEA BLOCK blocking reagent (Thermo Scientific) at room temperature for 1 h, followed by an overnight incubation with diluted antibody in SEA BLOCK buffer containing 0.2% Tween-20 at 4°C with gentle shaking. After washing with PBS containing 0.2% Tween-20, the membrane was incubated at room temperature for 1 h with either anti-rabbit IgG (H + L) DyLight 800 or anti-mouse IgG (H + L) DyLight 600 (Cell Signaling Technologies, Danvers, MA, United States). Blots were then visualized with Odyssey Infrared Imaging System (LI-COR Biosciences, Lincoln, NE, United States). Optical density measurements were taken using the Gel-Pro Analyzer software. Antibodies used were: p-Akt-S473 (#4060), Akt (#9272), p-mTOR (#5536S), mTOR (7C10), p-4E-BP1 (#236B4), 4E-BP1 (#53H11), OPA1 (#80471) from Cell Signaling Technologies, PDK4 (#ab214938), p-PDH (#ab177461), PDH (#ab168379), PGC1α (#ab3242) from Abcam, and α-Tubulin (#12G10) from Developmental Studies Hybridoma Bank (Iowa City, IA, United States).

### Real-Time Quantitative Polymerase Chain Reaction

RNA from gastrocnemius muscle was isolated using the miRNeasy Mini kit (Qiagen, Valencia, CA, United States), following the protocol provided by the manufacturer. RNA was quantified by using a Synergy H1 spectrophotometer (Biotek, Winooski, VT, United States). Total RNA was reverse transcribed to cDNA using the Verso cDNA kit (Thermo Fisher Scientific, Waltham, MA, United States). Transcript levels were measured by Real-Time PCR (Light Cycler 96, Roche, Indianapolis, IN, United States) taking advantage of the TaqMan gene expression assay system (Life Technologies, Carlsbad, CA). Expression levels for Atrogin-1 (Mm00499523_m1), Murf-1 (Mm01185221_m1), Fis1 (Mm00481580), Mfn (Mm00500120), PDK4 (Mm01166879), Pink1 (Mm00550827), Parkin (Mm00450187), Opa1 (Mm01349707), Pgc1α (Mm01208835), CytB (Mm04225271_g1), Lipe (Mm00495359), Pnpla2 (Mm00503040), Fasn (Mm00662319), Srebf1 (Mm00550338), Srebf2 (Mm01306292), Plin1 (Mm00558672_m1), Cpt1b (Mm.PT.6753512a1), Acaa1a (Mm.PT.18700004a1), Hadha (Mm.PT.33859811a1) were detected. Gene expression was normalized to TBP (Mm01277042_m1) levels using the standard 2^−ΔCT^ methods. The expression of TBP was not significantly altered in any of the experimental groups.

### Quantification of LDL Receptor in Skeletal Muscle

The levels of LDL receptor were measured in gastrocnemius skeletal muscle homogenates from all groups by using a specific ELISA kit (#MLDLR0; Bio-Techne Corporation, Minneapolis, MN, United States), according to the manufacturers protocol.

### Oil Red O Staining

For ORO staining, tibialis anterior muscles were sectioned (10 μm) and immediately fixed in ice cold formaldehyde (3.7%; Thermo Fisher Scientific) for 1 h. Sections were serially washed in Milli-Q water (MilliporeSigma) and stained in ORO working solution prepared as previously described (Huot et al., 2020a); for 45 min at room temperature in the dark. Following ORO staining, sections were again serially washed in Milli-Q water and then rinsed in running tap water for 10 min. Sections were mounted in 50% glycerol in PBS and photographed using an Axio Observer.Z1 motorized microscope (Zeiss). Entire ORO-stained sections were analyzed for signaling intensity and area of positive staining using ImageJ software.

### Enzymatic Activities

The enzymatic activities of hexokinase (HK), pyruvate dehydrogenase (PDH) and succinate dehydrogenase (SDH) were measured in gastrocnemius muscle homogenates using Colorimetric Assay Kits (#MAK091, MAK183 and #MAK197, respectively) from Sigma-Aldrich according to the manufacturer’s instruction. Briefly, 10 mg of skeletal muscle tissue was homogenized in 100 μL of ice-cold assay buffer and then centrifuged, and 10 μL of homogenate was added to 96-well plates. Appropriate reaction mix was added to each of the wells and the product of enzyme reaction, which results in a colorimetric (600 nm for SDH and 450 nm for HK and PDH) product proportional to the enzymatic activity. The absorbance was recorded by incubating the plate at 37°C taking measurements (600 or 450 nm) every 5 for 30 min.

### Statistical Analyses

Two-way analysis of variance (ANOVA) tests were performed to determine differences between experimental groups. *Post hoc* comparisons were accomplished *via* a Tukey’s test, with statistical significance set a priori at *p* ≤ 0.05. All statistics were performed using GraphPad Prism 8.4.1. Data are presented as means ± standard deviation.

## Results

### MitoQ Improves Myotube Atrophy and *in Vitro* Metabolism

**FIGURE 1:**
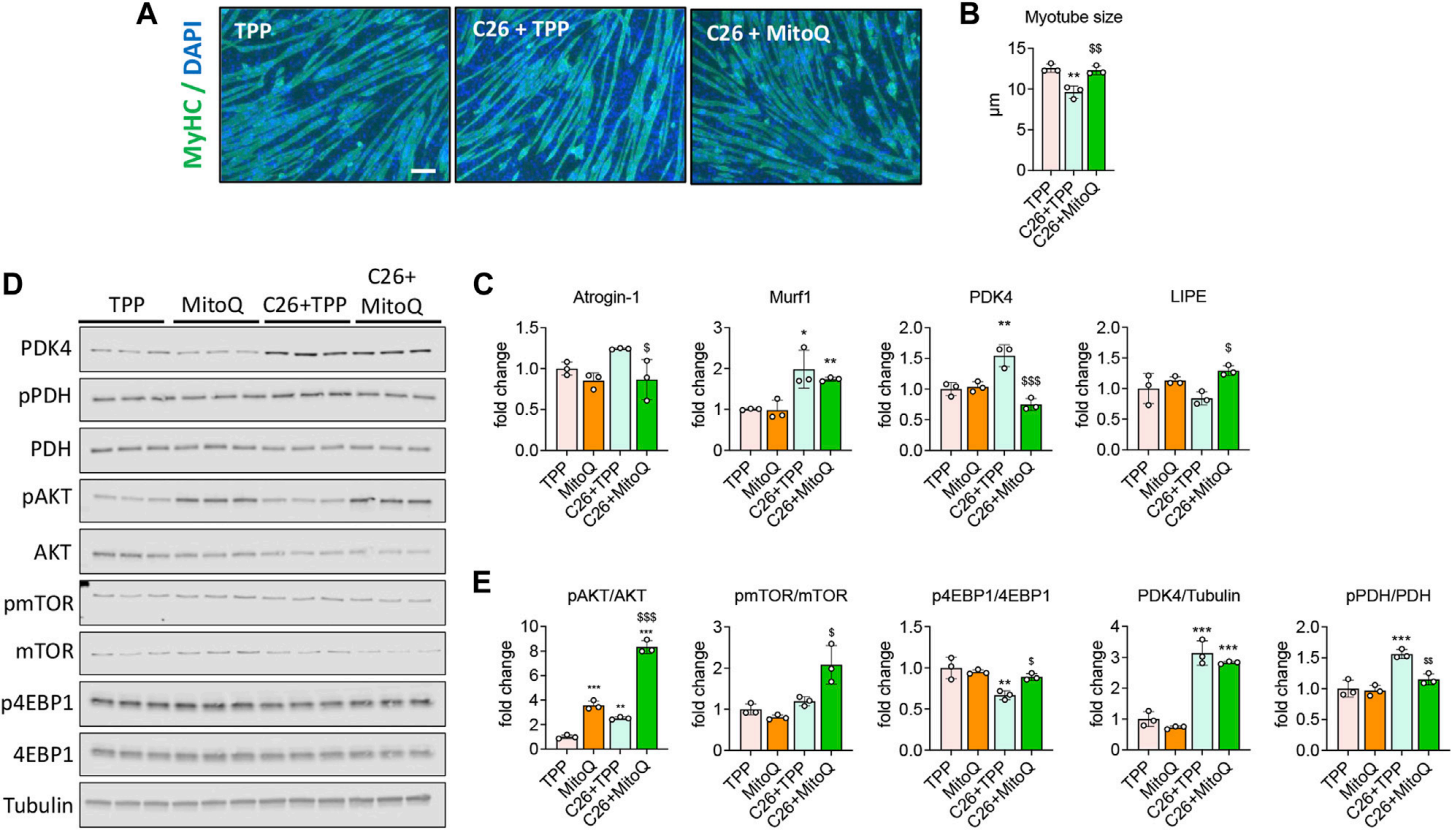
MitoQ improves myotube atrophy and *in vitro* metabolism. **(A,B)** Myosin heavy chain (MyHC; green) and DAPI (blue) immunofluorescent staining in C2C12 myotubes exposed to unconditioned medium or 20% C26 CM (C26) and treated with 75 µM MitoQ or TPP for up to 48 h. Myotube size was determined by measuring the minimum diameter of 250–350 myotubes per experimental condition (*n* = 3). Scale bar, 100 μm. **(C)** Gene expression levels for *Atrogin-1 Murf1, PDK4* and *Lipe* in C2C12 myotubes. Gene expression was normalized to *TBP* levels and expressed as fold change *vs.* TPP. **(D,E)** Representative Western blotting and quantification for phospho-AKT (pAKT), AKT, phospho-mTOR (pmTOR), mTOR, phospho-4EBP1 (p4EBP1), 4EBP1, PDK4, phospho-PDH (pPDH), PDH, expressed as fold change *vs.* TPP. Tubulin was used as loading control. Statistical significance was evaluated by two-way analysis of variance, and significant differences (at least *p* < 0.05) were reported as ^*^ *p* < 0.05, ^**^ *p* < 0.01, ^***^ *p* < 0.001 *vs.* TPP; ^$^ *p* < 0.05, ^$$^ *p* < 0.01, ^$$$^ *p* < 0.001 *vs.* C26 + TPP.

To clarify the *in vitro* effects of MitoQ in C2C12 myotube cultures exposed to tumor-derived conditioned medium (CM), fully differentiated C2C12 myotubes were administered 20% C26 CM and treated with MitoQ 75 μM, C26 + MitoQ) for 48 h. In line with our previous observations (Pin et al., 2019b), myotubes incubated with CM generated from C26 cells in combination with TPP showed reduced size when compared to TPP-treated controls (−23%, *p* < 0.01; Figure 1A). Interestingly, MitoQ protected the myotubes from atrophy induced by tumor-derived factors (Figures 1A,B). The muscle-specific ubiquitin ligase *Murf1* was increased after exposure to C26 CM (+98%, *p* < 0.001; Figure 1C), although the treatment was only able to prevent the overexpression of *Atrogin-1* (−30%, *p* < 0.001, Figure 1C). We then checked the expression of PDK4, involved in the regulation of the pyruvate dehydrogenase complex (PDH) and a key enzyme in energy metabolisms that we showed upregulated in the muscle of cachectic mice (Pin et al., 2019b). The myotubes exposed to C26 showed PDK4 increased at both mRNA and protein levels (+54% *p* < 0.01, and +213%, *p* < 0.001, respectively; Figures 1C–E), consistent with the increased phosphorylation of PDH (+56%, *p* < 0.01, Figures 1D,E). Administration of MitoQ reduced the levels of PDK4 gene expression, as well as the phosphorylation of PDH (−51% and −26%, *p* < 0.01, respectively; Figures 1C–E) in the C26 CM-treated myotubes. Since intramuscular lipid metabolism can be compromised during cachexia, we investigated the expression of Lipase E hormone sensitive (LIPE), known to be involved in intramuscular lipid accumulation (Haemmerle et al., 2002). *Lipe* was unchanged in the C26+TPP-treated myotubes*,* although MitoQ administration enhanced its expression (+53%, *p* < 0.05 *vs.* C26 + TPP; Figure 1C). To verify if the anabolic signaling was equally affected by the treatment, we assessed the phosphorylation of AKT and found it drastically increased by MitoQ administration (MitoQ: +2.5-fold, *p* < 0.001; C26 + MitoQ: +7-fold, *p* < 0.001 *vs.* TPP; Figures 1D,E). Similarly, the reduced anabolic signaling supported by the downregulation of p-4EBP1 (−33%, *p* < 0.01 *vs.* TPP) was corrected by the administration of MitoQ (+33%, *p* < 0.05 *vs.* C26 + TPP; Figures 1D,E).

### MitoQ Improves Body Weight, Muscle Mass and Weakness in C26 Hosts

**FIGURE 2:**
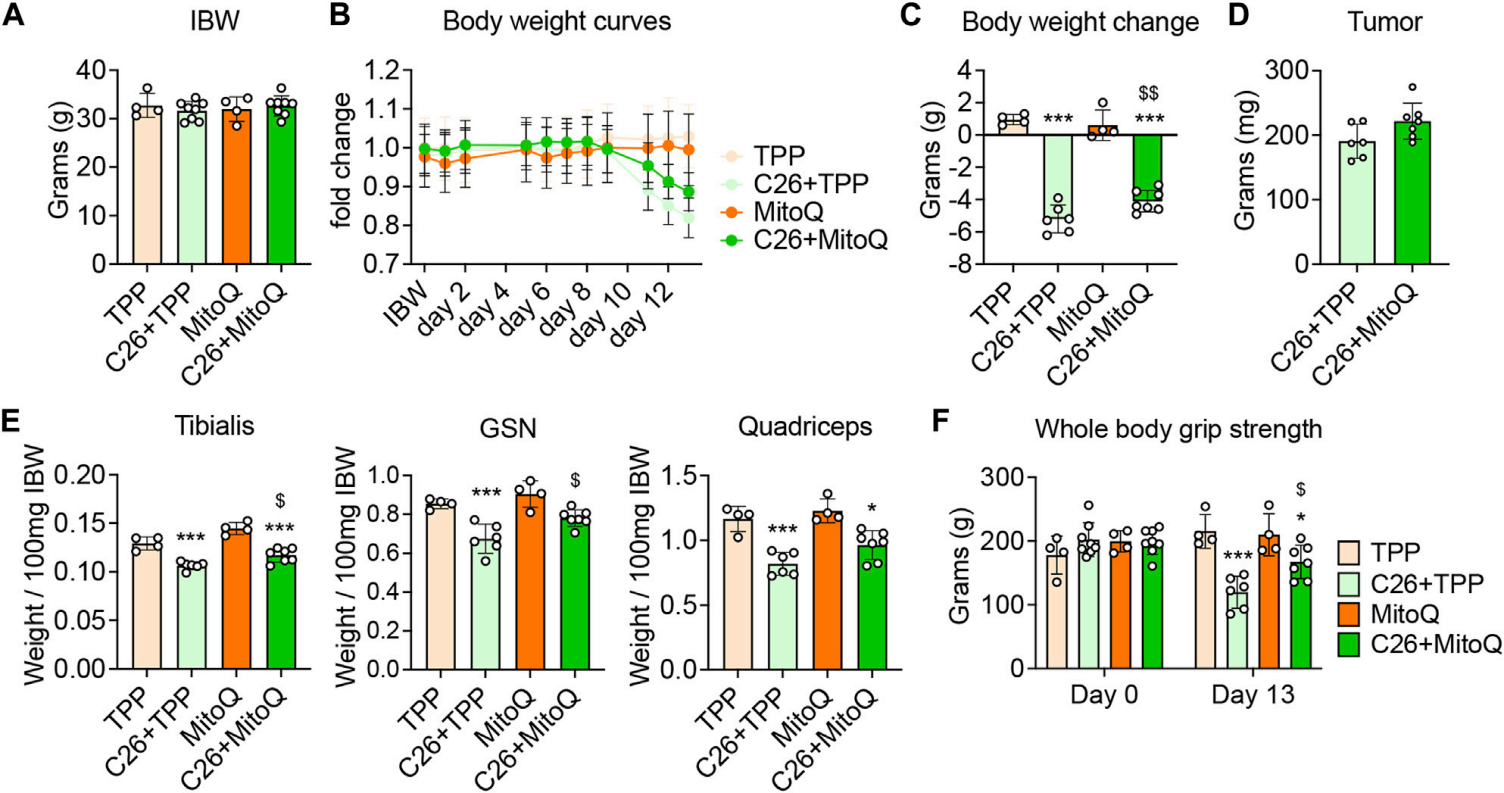
MitoQ treatment increases body weight in C26 hosts and partially corrects muscle mass and weakness. **(A)** Initial body weight (IBW) expressed in grams, recorded the day of tumor inoculation. **(B)** Body weight (BW) curves. **(C)** BW change at time of sacrifice (vs. IBW). **(D)** Tumor weight expressed in milligrams (mg). **(E)** Tibialis anterior, gastrocnemius (GSN) and quadricep muscle mass normalized to initial body weight (IBW) and expressed as weight/100 mg IBW. **(F)** Whole body grip strength (expressed in grams of force). Statistical significance was evaluated by two-way analysis of variance, and significant differences (at least *p* < 0.05) were reported as: ^*^ *p* < 0.05, ^***^ *p* < 0.001 *vs.* TPP; ^$^ *p* < 0.05, ^$$^ *p* < 0.01 *vs.* C26 + TPP.

To determine if MitoQ was sufficient to improve cancer cachexia, CD2F1 male mice were treated with MitoQ starting 2 weeks before the inoculum of C26 tumor cells and until the day of sacrifice. As shown in Figure 1A, body weights at time of tumor injection (*i.e.*, 11 weeks of age) were comparable across all experimental groups. The C26 + TPP hosts showed significant loss of body weight (BW) starting 9 days after tumor implantation (Figure 2B), whereas the administration of MitoQ was able to partially correct body wasting without interfering with tumor growth (Figures 2B–D). The C26 + TPP hosts lost skeletal muscle mass, as suggested by the reduced tibialis anterior (−17%, *p* < 0.001), gastrocnemius (−21%, *p* < 0.001) and quadriceps (−29%, *p* < 0.001) muscle mass (Figure 2E, Supplementary Figure S1A), along with the reduction of muscle strength (−45%, *p* < 0.001; Figure 2F, Supplementary Figure S1B) and muscle cross-sectional area (−9%, *p* < 0.05; Supplementary Figure S1C). Notably, the C26 + MitoQ group was partially protected from muscle mass loss, as supported by the increased size of the tibialis (+8%, *p* < 0.05) and gastrocnemius (+12%, *p* < 0.05) muscles (Figure 2E, Supplementary Figure S1A). Consistently, also muscle strength in the C26 hosts was improved by the treatment with MitoQ (+40%, *p* < 0.05; Figure 2F, Supplementary Figure S1B). Moreover, the spleen mass was severely increased in the C26 bearers independent of the treatment (+120%, *p* < 0.001 *vs.* TPP and +162%, *p* < 0.001 *vs.* MitoQ), whereas heart and liver were unchanged (Supplementary Figure S1D). Interestingly, the white adipose tissue (WAT) was severely depleted in the C26 + TPP hosts (−58%, *p* < 0.001; Supplementary Figure S1D), whereas it was 50% increased, though not significantly, in the tumor-bearing mice treated with MitoQ (*p* = 0.0675; Supplementary Figure S1D). For this reason, we decided to study the expression of the mitochondrial uncoupling protein-2 (UCP2), the most abundant UCP isoform in WAT, involved in the regulation of energy homeostasis (Pinkney et al., 2000). We found that MitoQ was able to reduce the expression of *UCP2* in WAT in the tumor-bearing mice (−59%, *p* < 0.05; Supplementary Figure S1E).

**FIGURE 3:**
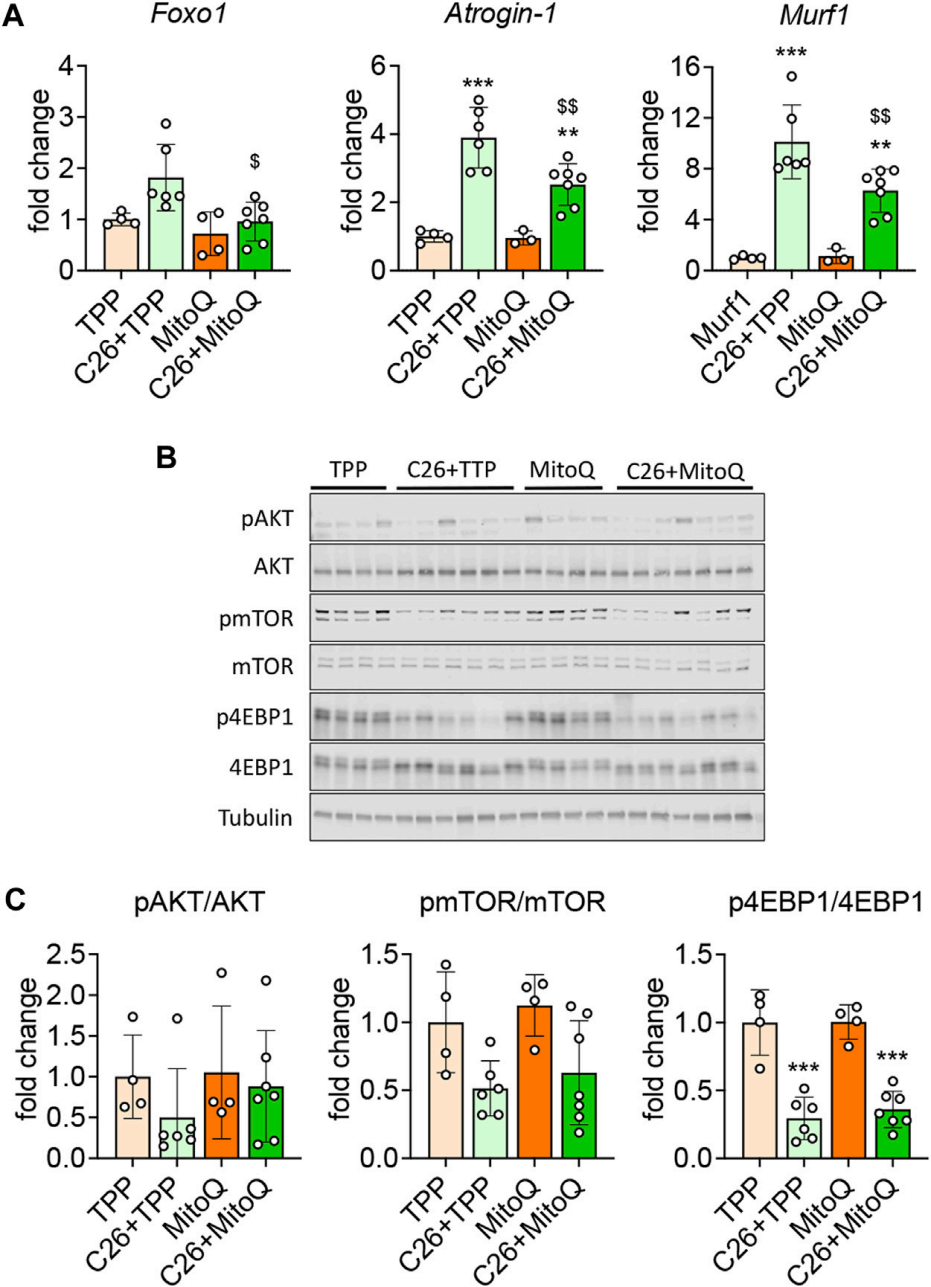
MitoQ treatment partially normalizes protein catabolism and anabolism. **(A)** Gene expression levels for *Foxo1*, *Atrogin-1* and *Murf1* in the gastrocnemius muscle. **(B,C)** Representative Western blotting and quantification for phospho-AKT (pAKT), AKT, phospho-mTOR (pmTOR), mTOR, phospho-4EBP1 (p4EBP1), 4EBP1. Protein expression was represented as a ratio of phosphorylated protein/total protein. Tubulin was used as loading control. Data were expressed as fold change *vs.* TPP. Statistical significance was evaluated by two-way analysis of variance, and significant differences (at least *p* < 0.05) were reported as ^**^ *p* < 0.01, ^***^ *p* < 0.001 *vs.* TPP; ^$^ *p* < 0.05, ^$$^ *p* < 0.01 *vs.* C26 + TPP.

As previously described (Huot et al., 2020a) and consistent with the reduced skeletal muscle mass, the mRNA expression of markers of protein catabolism such as *Atrogin-1* and *MuRF-1* was markedly increased in the muscle of C26 tumor hosts (+289%, *p* < 0.001; +912%, *p* < 0.001, respectively; Figure 3A) *vs.* TPP. Interestingly, the administration of MitoQ was able to partially revert the hypercatabolic state in the muscle of the C26 bearers, as shown by the reduced mRNA expression of *Atrogin-1* (−64%, *p* < 0.01), *MuRF-1* (−62%, *p* < 0.01), and *Foxo1* (−53%, *p* < 0.05; Figure 3A). Since skeletal muscle atrophy can be driven by an unbalance between protein degradation and synthesis (Bonaldo and Sandri, 2013), we investigated the expression of markers of anabolic signaling previously found altered in cancer cachexia (Huot et al., 2020a). As shown in Figures 3B,C, the p-AKT/AKT and p-mTOR/mTOR ratios were unchanged in all groups, whereas the p-4EBP1/4EBP1 ratio was reduced in the cachectic muscles (−70%, *p* < 0.001, respectively) with respect to the TPP-treated mice. These data suggest that, unlike our *in vitro* data (Figure 1), the treatment with MitoQ was unable to improve the anabolic signal, in line with unchanged AKT, mTOR or 4EBP1 phosphorylation levels (Figures 3B,C).

### MitoQ Improves Mitochondrial Homeostasis and Metabolism

**FIGURE 4:**
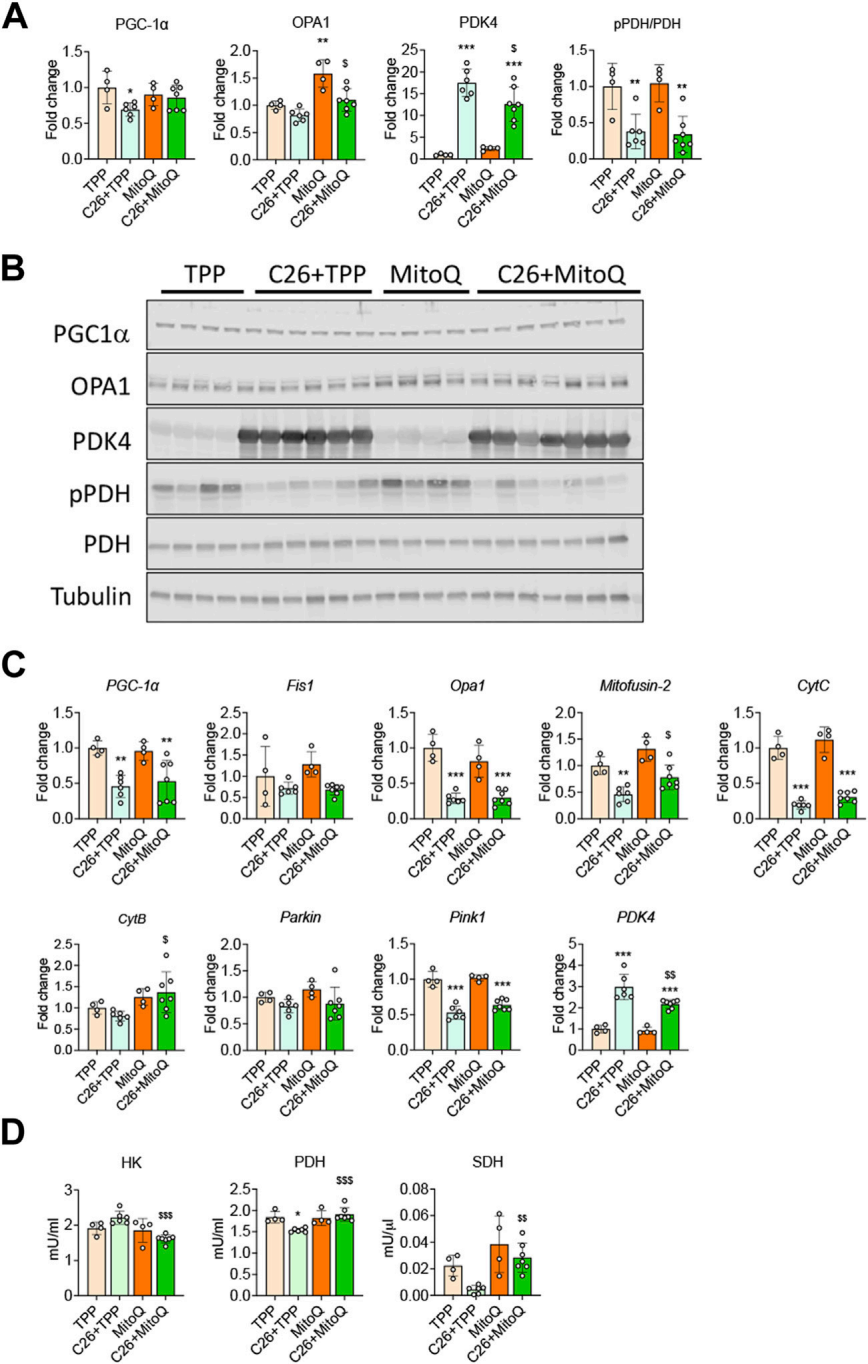
MitoQ affects mitochondrial homeostasis and metabolism. **(A,B)** Quantification and representative Western blotting for PGC1α, OPA1, PDK4, phospho-PDH (pPDH), PDH, expressed as fold change *vs.* TPP. Tubulin was used as loading control. **(C)** Gene expression levels for *PGC1α*, *Opa1, Fis1, Mitofusion-2, CytC, CytB, Parkin, Pink1* and *Pdk4* in the gastrocnemius muscle. Gene expression was normalized to *TBP* levels and expressed as fold change *vs.* TPP. **(D)** Muscle enzymatic activities of hexokinase (HK), pyruvate dehydrogenase (PDH) and succinate dehydrogenase (SDH) in the skeletal muscle were expressed in milliunits/mL (mU/mL) or milliunits/µL (mU/μL). Data are expressed as means ± SD. Statistical significance was evaluated by two-way analysis of variance, and significant differences (at least *p* < 0.05) were reported as: ^*^ *p* < 0.5 ^**^ *p* < 0.01 ^***^ *p* < 0.001 *vs.* TPP; ^$^ *p* < 0.05, ^$$^ *p* < 0.01, ^$$$^ *p* < 0.001 *vs.* C26 + TPP.

Since MitoQ acts directly within the mitochondria, we investigated markers of mitochondrial biogenesis, turnover and function. In line with our previous findings (Pin et al., 2019b; Huot et al., 2020a), the muscle of C26 bearers showed reduced protein and gene expression for PGC1α, one of the main regulators of mitochondrial biogenesis **(**−30%, *p* < 0.05 and −54%, *p* < 0.01, respectively; Figures 4A–C). The administration of MitoQ contributed to preserve PGC1α protein expression in the C26 hosts when compared to the TPP-treated hosts (−47% *p* < 0.05 and Figures 4A,B). With regards to the markers of mitochondrial dynamics and mitophagy, the mRNA expression levels of *Fis1* and *Parkin* were unchanged across all groups (Figure 4C), whereas the regulators of mitochondrial fusion and fission *OPA1*, *Mitofusin-2* and *Pink1* were downregulated in the muscle of C26 + TPP hosts. Interestingly, only the expression of *Mitofusin-2* was significantly improved by the treatment (+70%, *p* < 0.05; Figure 4C). As shown in Figures 4A,B, OPA1 protein levels were not significantly affected by the growth of the C26 tumor, whereas MitoQ was able to upregulate the expression of OPA1 in the muscle of both healthy and tumor-bearing mice (Figures 4A,B). To clarify if the oxidative function was improved using mitochondria-targeting agents we analyzed the gene expression of the mitochondria-specific enzymes Cytochrome C (CytC) and Cytochrome B (CytB) and found that only the former was significantly reduced in the muscle of the tumor-bearing mice, whereas MitoQ did not provide any protective effect (Figure 4C). In line with our *in vitro* data, PDK4 was increased at both gene and protein levels in the tumor hosts, whereas MitoQ partially prevented PDK4 overexpression (Figures 4A–C). Moreover, PDH phosphorylation was not increased in the C26 model and MitoQ treatment had no effect in any of the groups (Figures 4A,B). To evaluate if the changes observed at molecular levels were also followed by modulation of the energetic metabolism, we assessed the activity of key enzymes, including HK, PDH and SDH. As shown in Figure 4D, HK and SDH enzymatic activities were unchanged in the muscle of C26 + TPP mice, whereas the treatment with MitoQ reduced HK (−27%, *p* < 0.001) and increased SDH (+80%, *p* < 0.01; Figure 4D) in the presence of C26 tumors. Finally, consistent with the expression of PDK4, the activity of PDH was markedly reduced in the cachectic muscles (−17%, *p* < 0.05) and corrected by administration of MitoQ (+24%, *p* < 0.001; Figure 4D).

### MitoQ Treatment Reduces Myosteatosis and Improves β-Oxidation

**FIGURE 5:**
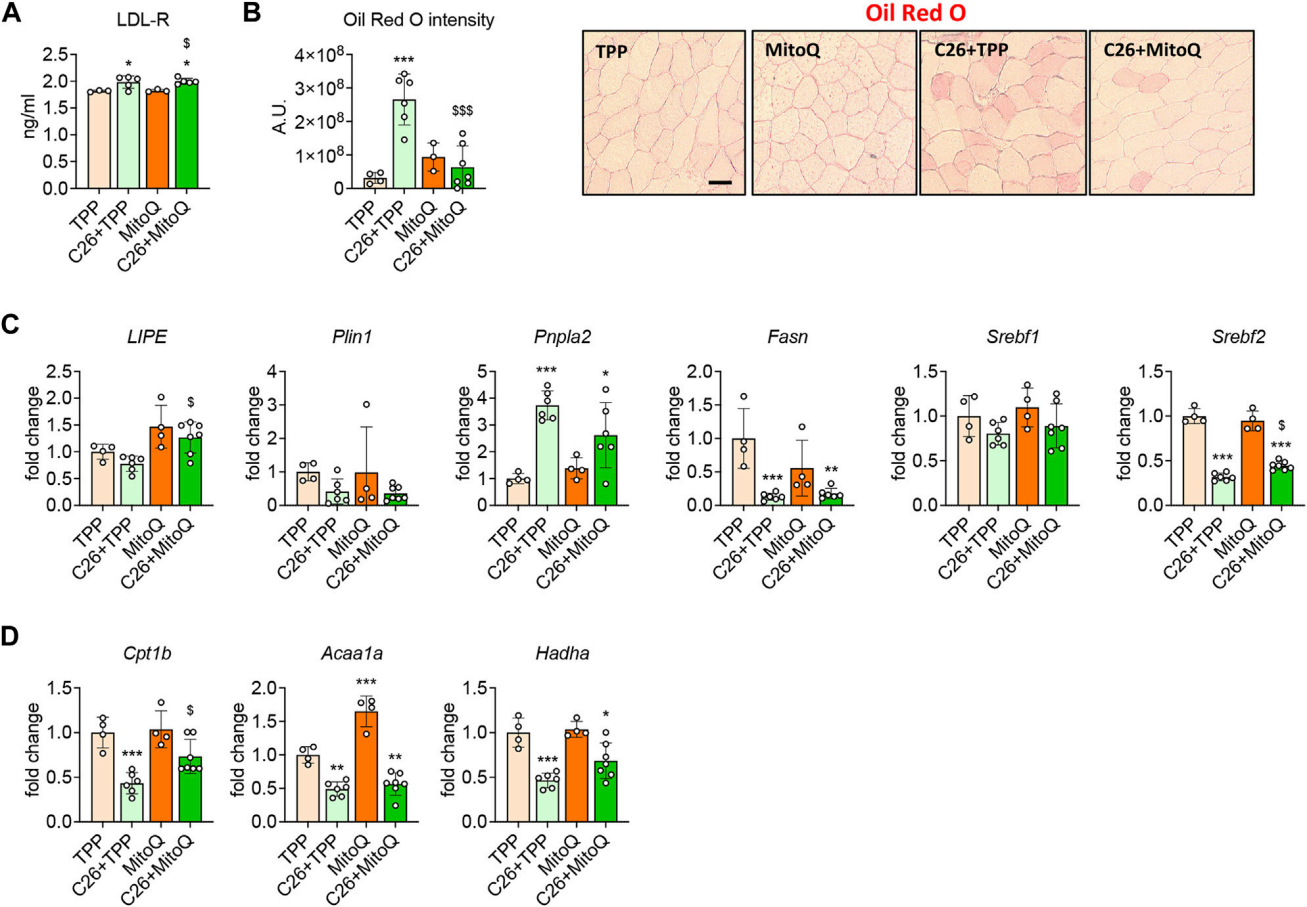
MitoQ treatment reduces myosteatosis and improves β-oxidation. **(A)** Quantification of LDL-R levels in gastrocnemius muscle. **(B)** Oil Red O staining and quantification of tibialis anterior muscle. Scale bar, 100 μm. **(C)** Gene expression levels for markers of lipolysis and lipogenesis such as *Lipe, Plin1, Pnpla2, Fasn, Srebf1* and *Srebf2* in the gastrocnemius muscle. **(D)** Gene expression levels for markers of β-oxidation such as *Cpt1b, Acaa1a* and *Hadha* in the gastrocnemius muscle. Gene expression was normalized to *TBP* levels and expressed as fold change *vs.* TPP. Data are expressed as means ± SD. Statistical significance was evaluated by two-way analysis of variance, and significant differences (at least *p* < 0.05) were reported as: ^*^ *p* < 0.5 ^**^ *p* < 0.01, ^***^ *p* < 0.001 *vs.* TPP; ^$^ *p* < 0.05, ^$$$^ *p* < 0.001 *vs.* C26 + TPP.

Abnormal lipid metabolism, as suggested by hyperlipidemia, elevated levels of LDL-R (Pin et al., 2019a) and increased myosteatosis (Huot et al., 2020a) characterize the C26 models and could reflect altered utilization of fat as energy substrate. Hence, we investigated whether the use of MitoQ could improve these alterations. Similar to our previous study (Pin et al., 2019a), the levels of LDL-receptor (LDL-R) were increased in the muscle of cachectic hosts **(**+10%, *p* < 0.05; Figure 5A), in line with the elevated intramuscular fat accumulation, as measured by Oil Red-O in the tibialis anterior transversal sections from C26 tumor-bearing mice (Figure 5B). Interestingly, MitoQ treatment did not modulate the expression of LDL-R (Figure 5A), although it was able to decrease fat accumulation in the skeletal muscle (Figure 5B). To investigate the molecular mechanisms subtending to the changes observed in fat accumulation we assessed the expression of markers of lipogenesis and lipolysis in the skeletal muscle of control and tumor hosts. The levels of *Lipe*, a regulator of lipolysis, as well as of *Plin1* and *Srebf1*, involved in the control of lipid metabolism, were unchanged in the tumor hosts (Figure 5C). Conversely, the regulators of lipogenesis *Fasn* and *Srebf2* were markedly reduced (−87% and −68%, *p* < 0.001, respectively) and *Pnpla2* was increased (+273%, *p* < 0.001; Figure 5C). Upon MitoQ administration, *Lipe* and *Srebf2* were found significantly increased in the skeletal muscle of C26 bearers (+63% and +40%, *p* < 0.05, respectively; Figure 5C). Since the changes in intramuscular fat accumulation could be associated with altered utilization of fat as energy substrate, markers of β-oxidation were also measured. The gene expression for *Cpt1b*, *Acaa1a* and *Hadha* was reduced in the cachectic muscle (−57%, −45% and −53%, *p* < 0.01, respectively; Figure 5D), whereas only *Cpt1b* was positively regulated by the treatment (+64%, *p* < 0.05; Figure 5D).

## Discussion

Cancer cachexia is a complex, multifactorial syndrome characterized by functional and metabolic deficits affecting multiple tissues and organs. No approved treatments are currently available for cachexia in the US. We and others have contributed to highlight that cachexia induced by cancer promotes metabolic perturbations in several organs (Pin et al., 2019a), and these can even appear ahead of any appreciable reduction in body weight (O'Connell et al., 2021). In particular, changes in the energetic status seem to play a pivotal role in driving the functional impairments typically observed in cancer patients. In such regard, the negative energy balance seems to be tightly interconnected with the appearance of mitochondrial alterations (Fearon et al., 2011). A direct connection between altered mitochondrial metabolism and skeletal muscle atrophy has been previously reported (Pin et al., 2019b), also supporting several studies aiming to investigate the potential of mitochondria-targeted strategies for the therapy of cachexia. Here we investigated whether the use of MitoQ was able to improve the cachectic phenotype induced by the C26 tumor, a well-characterized preclinical model for the study of cancer cachexia.

MitoQ is a mitochondria-targeting antioxidant and dietary supplement that can be orally administered and is well tolerated in a long-term use in both humans and animals (Murphy, 2008; Snow et al., 2010; Shill et al., 2016). It is composed by the antioxidant portion ubiquinol, which is oxidized in ubiquinone and rapidly reduced by the complex II to restore the antioxidant capacity, and the TPP^+^ cation portion, which in turn allows its accumulation within the mitochondrial inner membrane (Broome et al., 2018). Among the mechanisms of action of MitoQ, prevention of lipid peroxidation, as well as peroxynitrile, superoxide and protein oxidation, represent the most characterized (Murphy, 2008; Zinovkin and Zamyatnin, 2019).

Several preclinical and clinical studies using MitoQ revealed its potential to improve different pathological states. For example, it has been shown that MitoQ can improve endothelial function and reduce oxidative stress in healthy older adults (Rossman et al., 2018), whereas its administration was beneficial in reducing alcoholic hepatosteatosis in mice (Chacko et al., 2011), as well as in decreasing liver damage induced by chronic HCV infection in humans (Gane et al., 2010). MitoQ treatment also improved mitochondrial function in a rat model of pressure overload and in a rodent model of endotoxin-induced cardiac dysfunction (Supinski et al., 2009; Ribeiro Junior et al., 2018). Contrarily, the skeletal muscle loss and function as well as the oxidative damage were not improved by MitoQ in an aging murine model (Sakellariou et al., 2016), and no beneficial effects were detected in a human clinical study conducted in subjects with Parkinson’s disease (Snow et al., 2010), despite previous promising *in vitro* observations (Mao et al., 2013).

Here we showed that MitoQ treatment was able to counteract myotube atrophy in C2C12 cultures exposed to tumor-derived factors. Our data are in line with previous observations supporting the ability of this compound to prevent the loss of myosin content in C2C12 myotubes exposed to the chemotherapeutics doxorubicin and taxol (Guigni et al., 2018). These beneficial effects were also confirmed in our *in vivo* experiments. Indeed, MitoQ treatment was able to improve the cachectic phenotype induced by the C26 tumor growth. In particular, the body weight was maintained in the tumor-bearing mice receiving MitoQ, consistent with previous data generated in C26 hosts administered SS-31, another mitochondria-targeted compound (Ballaro et al., 2021), and with the partial preservation of skeletal muscle mass in our model. Interestingly, and in contrast with our data, the SS-31 was unable to improve muscle mass in the C26 tumor-bearing mice (Ballaro et al., 2021), despite being previously reported to prevent myofiber atrophy induced by limb immobilization (Min et al., 2011). Consistent with the preservation of muscle mass we also found improved muscle strength following MitoQ treatment in the C26 bearers, in agreement with evidence that the SS-31 treatment was sufficient to improve the whole-body strength in the tumor hosts receiving chemotherapy (Ballaro et al., 2021).

The mechanisms by which MitoQ protects skeletal muscle mass are partially unknown. Muscle wasting during cancer cachexia often results from overactivation of protein catabolism, mainly by hyperactivation of the ubiquitin-proteasome system (UPS) (Bonaldo and Sandri, 2013; Rom and Reznick, 2016). Several studies using both human and rodent samples have contributed to establish the increased expression of muscle-specific E3 ligases as markers of UPS activation (Rom and Reznick, 2016). In our *in vitro* and *in vivo* studies, we showed partially corrected expression of the E3 ligases Atrogin-1 and Murf1 upon MitoQ administration, suggesting that this could be one of the mechanisms of action by which the mitochondrial antioxidant preserves muscle mass in cancer. These observations are also consistent with previous findings reporting the negative modulation of E3 ligases in C2C12 myotubes exposed to the protonophore carbonyl cyanide m-chlorophenylhydrazone (CCCP) in combination with MitoQ (Lee et al., 2020).

Altered protein anabolism has also been reported to participate in the negative nitrogen balance that characterizes the cachectic muscle (Tessitore et al., 1993; Kim et al., 2021), and this alteration can be detected even before the activation of protein degradation (Svaninger et al., 1983). One of the major pathways regulating protein anabolism in skeletal muscle is the AKT-mTORC pathway (Schiaffino et al., 2021). In our *in vitro* study, MitoQ treatment strongly enhanced the activation of the AKT-mTORC pathway in myotubes exposed to CM. Contrarily, our *in vivo* experiment did not show any correction of the muscle anabolic signaling upon MitoQ administration, likely due to the variety of pro-atrophic stimuli in a whole organism. Altogether this data suggest that the use of mitochondria-targeted agents improves muscle atrophy at least in part by correcting the unbalance between protein catabolism and anabolism. Future studies performed in different preclinical cancer cachexia models will need to validate these observations.

Loss of adipose tissue is amongst the hallmarks of cachexia (Fouladiun et al., 2005; Das et al., 2011). In our *in vivo* experiment, we found that tumor hosts administered MitoQ displayed a marked, though not statistically significant, sparing of WAT, thus prompting us to investigate whether the energy metabolisms in fat could be modulated as well. Our data did not reveal changes in the expression of UCP1, a marker of WAT browning involved in the thermogenic waste of energy during cancer cachexia (Bing et al., 2000), whereas UCP2 levels were reverted by MitoQ treatment. Notably, UCP2 expression was previously shown to be inversely related to adiposity (Oberkofler et al., 1998; Pinkney et al., 2000) and was found elevated in conditions of high reactive oxygen species (Patterson et al., 2012), likely functioning as a protective mechanism against oxidative damage (Krauss et al., 2003). Altogether, these observations would seem to corroborate the idea that MitoQ preserves adiposity in C26 bearers mainly by means of its antioxidant properties.

We have described that several factors related with mitochondrial biogenesis, turnover and function were downregulated in skeletal muscle in several preclinical mouse models of cancer (Pin et al., 2018; Huot et al., 2020a; Huot et al., 2020b), thus suggesting that the mitochondrial environment could be a key target for therapeutic intervention in cachexia. Despite the fact that most of the mitochondrial factors investigated in the present study were abnormally modulated in the skeletal muscle of C26 hosts, only the expression of Mitofusin-2 and CytB was increased by the treatment with MitoQ. Our observations were corroborated by recent data confirming the ability of MitoQ to increase Mitofusin-2 expression in C2C12 cells (Lee et al., 2020), as well as in the myocardial muscle during heart failure (Kim et al., 2020). Interestingly, MitoQ was also able to correct the overexpression of PDK4, although it was only partially effective in the *in vivo* setting. As we showed in a recent study, this kinase is an important negative regulator of PDH and, consequently of the TCA cycle, and its overexpression is sufficient to cause muscle atrophy (Pin et al., 2019b). In line with these findings, the phosphorylation of PDH was reduced in atrophic C2C12 myotubes exposed to MitoQ, nicely reflecting the improvement of mitochondrial metabolism as supported by the modulation of PDH and SDH activities *in vivo*. Also in this case, our data are consistent with evidence showing the ability of SS-31 to increase SDH activity and ATP production in the muscle of mice bearing C26 tumors (Ballaro et al., 2021). Altogether, these sets of data highlight the ability of MitoQ to modulate the oxidative metabolism in tumor hosts.

We previously showed evidence of systemic increased glucose demand in the C26 model, which correlated with an oxidative-to-glycolytic shift in fiber type and with increased HK activity (Pin et al., 2019a; Huot et al., 2020a). Conversely, β-oxidation was found significantly reduced at systemic level in the C26 hosts (Pin et al., 2019a). Findings from preclinical animal models and clinical studies showed high levels of glycerol, free fatty acid, triglycerides, and LDL particles in plasma during cachexia (Gercel-Taylor et al., 1996; Pin et al., 2019a; Riccardi et al., 2020), likely resulting from the adipose tissue wasting (Dalal, 2019). In this context, the unchanged levels of LDL-R and the reduced intramuscular fat accumulation suggest that MitoQ is somewhat able to improve the utilization of lipids as a major source of energy. This observation is corroborated by the increased expression of *Lipe* and *Cpt1b* in the muscle of MitoQ-administered mice, suggesting that lipolysis and β-oxidation are enhanced by the treatment.

Overall, our study demonstrates that MitoQ treatment can partially correct the cachectic phenotype induced by the C26 colorectal tumor. Our macroscopic observations showing improved muscle mass and strength are correlated by evidence of normalization of muscle energy metabolism following administration of MitoQ, in line with reduced HK activity and improved TCA cycle, overall suggesting a reversal of the oxidative-to-glycolytic metabolic shift that normally characterizes the cachectic muscles. Moreover, our data suggest that MitoQ could enhance the utilization of lipids accumulated within the skeletal muscle, thus contributing to rebalance the energy metabolism.

Despite these promising observations, we are aware of some limitations of our study. For example, our experimental approach conducted in a preclinical model of cancer cachexia included a 2-weeks pretreatment with MitoQ prior to tumor inoculation, hence hardly resembling the clinical setting. In order to validate MitoQ as an anti-cachexia therapeutic strategy, further investigations will need to determine its ability to counteract muscle wasting in rodents that already present evidence of ongoing cachexia. Importantly, MitoQ was previously investigated as dietary supplement in both long-term preclinical and clinical studies and revealed high safety profiles and limited toxicities. Hence, future investigations will be targeted at testing MitoQ beneficial anti-cachectic properties in healthy subjects and cancer patients receiving chronic administrations of the drug (Murphy, 2008; Snow et al., 2010; Shill et al., 2016). Lastly, in our study we failed to report on the antioxidant properties of MitoQ in the skeletal muscle of tumor hosts. Keeping in mind our previous observations demonstrating that chemotherapy, similar to cancer, also promotes mitochondrial abnormalities and higher levels of reactive oxygen species, which in turn contribute to the development of skeletal muscle dysfunction in cachexia (Barreto et al., 2016a; Barreto et al., 2016b; Brown et al., 2017; Ballaro et al., 2021; Beltra et al., 2021), future studies will need to validate the anti-cachectic properties of MitoQ also in combination with routinely administered anticancer agents.

## Data Availability

The raw data supporting the conclusion of this article will be made available by the authors, without undue reservation.
