# Supplementary material for: The Mitochondria-Targeting Agent MitoQ Improves Muscle Atrophy, Weakness and Oxidative Metabolism in C26 Tumor-Bearing Mice
Source: Front Cell Dev Biol. 2022 Mar 22;10:861622. doi: 10.3389/fcell.2022.861622 (PMC8980422; doi:10.3389/fcell.2022.861622)
Supplement: Supplementary file 1 [file DataSheet1.pdf]

The mitochondria-targeting agent MitoQ improves muscle atrophy, weakness and oxidative metabolism in C26 tumor-bearing mice

Fabrizio Pin, Joshua R. Huot, Andrea Bonetto

Supplementary Information

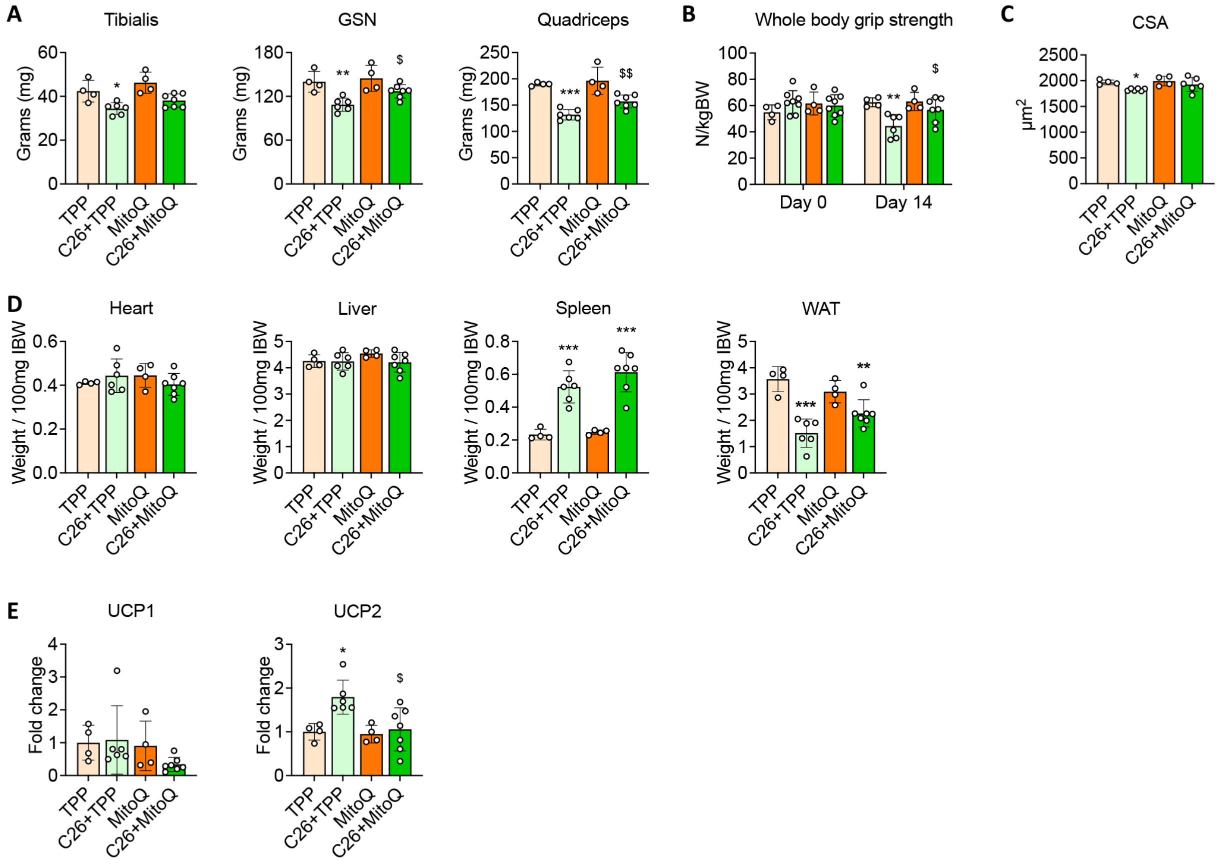

Figure S1. **A:** Tibialis anterior, gastrocnemius (GSN) and quadriceps muscle weights (expressed in mg). **B:** Whole body grip strength expressed in Newtons/kg body weight (N/kgBW). **C:** cross sectional area (CSA) in tibialis anterior muscle (expressed in μm^2). **D:** Heart, liver, spleen and white adipose tissue (WAT) mass normalized to initial body weight (IBW) and expressed as weight/100 mg IBW. **E:** Gene expression levels for *UCP1* and *UCP2* in WAT. Gene expression was normalized to *TBP* levels and expressed as fold change *vs.* TPP. Statistical significance was evaluated by two-way analysis of variance and significant differences (at least $P < 0.05$) were reported as: $^{*}p<0.05$, $^{**}p<0.01$, $^{***}p<0.001$ *vs.* TPP; $^{\$}p<0.05$, $^{\$\$}p<0.01$ *vs.* C26+TPP.
